# Sensitivity to heat in MS patients: a factor strongly influencing symptomology - an explorative survey

**DOI:** 10.1186/1471-2377-11-27

**Published:** 2011-02-25

**Authors:** Gullvi Flensner, Anna-Christina Ek, Olle Söderhamn, Anne-Marie Landtblom

**Affiliations:** 1 Department of Medicine and Health, Division of Nursing Science, Faculty of Health Sciences, Linköping University, SE-581 85 Linköping, Sweden; 2 Department of Nursing, Health and Culture, University West, SE-461 86 Trollhättan, Sweden; 3 Centre for Caring Research-Southern Norway, Faculty of Health and Sport Sciences, University of Agder, PO Box 509, NO-4898 Grimstad, Norway; 4 Division of Neuroscience, Department of Clinical and Experimental Medicine, Faculty of Health Sciences, Linköping University, SE-581 85 Linköping, Sweden; 5 Neurology Clinic, University Hospital, SE-581 85 Linköping, Sweden; 6 Neurology Unit, General Hospital, SE-591 85 Motala, Sweden

## Abstract

**Background:**

Many individuals diagnosed with Multiple Sclerosis (MS) are sensitive to increased body temperature, which has been recognized as correlating with the symptom of fatigue. The need to explore this association has been highlighted. The aim of this study was to investigate the occurrence of heat sensitivity and its relations to disease course, disability, common MS-related symptoms and ongoing immunosuppressive treatments among individuals 65 years of age or younger diagnosed with MS.

**Methods:**

A cross-sectional designed survey was undertaken. A questionnaire was sent to MS-patients with an Expanded Disability Status Score (EDSS) in the interval of 0-6.5 and who were between 20 and 65 years of age, living in an eastern region of Sweden (n = 334). Besides occurrence of heat sensitivity (Yes/No) and corresponding questions, the Fatigue Severity Scale (FSS), the MS-related symptom checklist and the Perceived Deficit Questionnaire (PDQ) were included. Data were analysed in relation to data level using Chi-square, Mann Whitney U-test, and Student's t-test. Pearson's and Spearman's correlations were calculated. In the logistic regression analyses (enter) dichotomized MS-symptoms were used as dependent variables, and EDSS, disease-course, time since onset, heat-sensitivity, age and sex (female/male) were independent variables. In the linear regression analyses, enter, mean FSS and summarized PDQ were entered as dependent variables and EDSS, disease-course, time since onset, heat sensitivity, age and sex (female/male) were independent variables.

**Results:**

Of the responding patients (n = 256), 58% reported heat sensitivity. The regression analyses revealed heat sensitivity as a significant factor relating not only to fatigue (p < 0.001), but also to several other common MS symptoms such as pain (p < 0.001), concentration difficulties (p < 0.001), and urination urgency (p = 0.009).

**Conclusions:**

Heat sensitivity in MS patients is a key symptom that is highly correlated with disabling symptoms such as fatigue, pain, concentration difficulty and urination urgency.

## Background

Between 60 and 80% of individuals diagnosed with the neurological disease Multiple Sclerosis (MS) have been reported as being sensitive to environmental heat [1]. In a multinational Internet-based survey of MS patients (n = 2 529), 70% reported that high temperatures worsened their MS [2]. Clinically, increased body temperature can result in increased neurological signs and MS symptoms. Blurred vision, known as Uthoff's phenomenon and first described in 1890, is caused by increased body temperature due to physical exercise or physical restraint [3]. The body temperature is found to influence nerve impulses, which are blocked or slowed down in a damaged nerve [4-6]. After normalization of the temperature, signs and symptoms improve or disappear [1,3].

Heat sensitivity has been described as a significant correlate of the symptom of fatigue in MS [5,7-9]. Together with divided attention and reduced muscular endurance, both heat sensitivity and fatigue have been reported as predictors of accidental falls [10]. Recently, Marino [6] stressed that heat sensitivity has been disregarded in studies focusing on fatigue.

In studies covering several decades, the occurrence rate of fatigue among individuals with MS is described as varying from 60 to more than 90% [5,11-13]. Irrespective of disease course, already in 1984 Freal and co-workers [5] reported fatigue as the very first symptom in about a third of patients. This initial symptom has been found to persist over the disease trajectory [14,15]. None of these studies, however, has focused on heat sensitivity.

During recent decades, individuals diagnosed with MS have had increased opportunities for treatment through the development of immune-modulating medications. These products affect the frequency of relapses and slow the progression of disability. Some immune-modulating products (e.g. beta-interferon) have side effects of influenza-like symptoms and fatigue, and may also increase depressive symptoms [16]. However, in treatment with beta-interferon, the initial side effects of influenza-like symptoms and perceived fatigue often decrease when the treatment spans a longer period. Another product, glatiramer acetate, is clinically considered neutral in this respect [17]. A relatively new immune-modulating product (natalizumab) has been reported to decrease the symptom of fatigue [18].

The aim of this study was to investigate the occurrence of heat sensitivity and its relations to disease course, disability, common MS-related symptoms (especially fatigue), and ongoing immunosuppressive treatments among individuals 65 years of age or younger diagnosed with MS and living in an eastern county of Sweden.

## Methods

### Study group

In order to reach individuals diagnosed with MS living in the area, a cross-sectional designed survey was undertaken, addressed to the individuals registered in the Swedish MS register (SwMS-reg). Together with information about the study, a questionnaire and a pre-paid reply envelope were sent to 334 individuals fulfilling the following criteria: i) being diagnosed with MS, ii) having an Expanded Disability Status Score (EDSS) [19] in the interval 0≤EDSS≤6.5, and iii) being of working age, i.e. 20-65 years, as 65 is the official retirement age. The questionnaires were distributed during 2007 to individuals with EDSS 1-6.5, and a complementary distribution was sent to individuals with EDSS = 0 in 2008. To those who had not answered within three weeks, one reminder was sent.

### Data collection

The individual's age, sex, disease course, i.e. relapsing-remitting (RR), secondary progressive (SP), primary progressive (PP) and function, measured with the EDSS [19], together with date of onset of MS, were obtained from the SwMS register. The diagnostic criteria used in the SwMS register are both Poser criteria [20] (older cases) and McDonald criteria [21] (recent time). Secondary progressive course of MS (SP) is a clinical neurological status whose onset can be hard to pinpoint. In clinical trials and other studies, this status is often not defined in detail. In the SwMS register the following definition is chosen: A phase of the disease where one can secure a slowly increasing pyramidal para- or even tetraparesis, sometimes also a bladder syndrome. In some cases can it be a cerebellar syndrome. A crucial criterion is that you observe increasing pyramidal (or cerebellar) symptoms without any signs of bouts [22].

The patients' EDSS and disease-course are continuously upgraded at physicians call at the neurological policlinics. For the majority of the participants in this study, both the EDSS and disease-course had been determined within a year before the data collection started. Common background factors, such as civil status, family, level of education and ongoing medical treatment, were requested in the questionnaire. Occurrence of heat sensitivity was asked about in a single question, "Are you sensitive to heat?" (Yes/No), with follow-up questions concerning how this was experienced when in sunshine, in a warm room, or taking a hot bath or shower, and what room-temperature the patient preferred. Occurrence and perceived severity of fatigue were requested as well. Furthermore, the following instruments were included: the Fatigue Severity Scale (FSS) [7,11]; the MS-related symptom checklist [23] and the Perceived Deficit Questionnaire (PDQ) [24].

In this study, and based on earlier studies [12,14,25], the EDSS was grouped according to normal neurological condition (EDSS = 0), mild disability (1.0≤EDSS≤3.5), moderate disability (4.0≤EDSS≤5.5) and severe disability (6.0≤EDSS≤6.5).

The FSS [7,11] comprises nine items covering perceived severity of fatigue, and each item is graded from least fatigue (1) to severe fatigue (7). As in earlier studies [e.g. [9,14], in this study an FSS mean score ≤4 was regarded as indicating no fatigue, >4 but <5 borderline fatigue, and ≥5 severe fatigue. The FSS has been used in earlier studies in Sweden [14] and is also used clinically. In this study, concurrent validity was assessed through correlations between the FSS with questions about the impact of fatigue on daily life as well as the occurrence of fatigue, rated on the MS-related symptom checklist [23], and resulted in *r *= 0.79, (*P *< 0.01) and *r *= 0.77 (*P *< 0.01), respectively. Reliability was assessed using Cronbach's alpha, and the alpha coefficient was 0.93.

The MS-related symptom checklist [23] was used to report 25 common MS symptoms such as fatigue, weakness in arms and legs, balance problems, pain, numbness, blurred vision, depression and difficulties with urination such as frequency and urgency. The occurrence of each symptom was graded in six steps from never (1) to always (6), which were then dichotomized, into never/sometimes (1-3) to 1 and usually/always (4-6) to 2. The checklist has been found to be closely related to the EDSS [23], and has been used in an earlier study in Sweden [13].

Cognitive dysfunction was assessed using the PDQ [24], measuring perceived problems with memory, attention and concentration. The original English version of the PDQ was translated into Swedish and then back-translated in accordance with Streiner and Norman [26], with good agreement. Twenty items are graded from never (0) to always (4). Summated scores vary from 0 to 80, where higher scores indicate greater cognitive problems. In this study, concurrent validity, assessed through correlations between the PDQ sum score and the MS-related symptoms forgetfulness and concentration difficulties, was r = 0.75 (*P *< 0.010) and r = 0.73 (*P *< 0.010), respectively. Reliability was tested using Cronbach's alpha (α = 0.95) and the split-half technique (r = 0.87).

### Statistical analyses

The data have been treated and analysed in relation to perceived heat sensitivity. The descriptive statistics used are in congruence with measurement scale level, except for the FSS which is treated on interval level as in earlier studies [e.g. [9,11,14]. Differences between groups have been tested using the chi-square test on data on nominal level, the Mann Whitney U-test on data on ordinal level, and Student's t-test on data on interval level. To test associations between variables, Pearson's and Spearman's correlations were calculated. In the logistic regression analysis (enter), MS symptoms that were significantly more frequent among heat-sensitive participants were dichotomized and entered as dependent variables, and EDSS (0-6.5), disease course (relapsing-remitting/progressive forms), heat sensitivity (yes/no), time since onset, and age and sex (female/male) were independent variables. In the linear regression analysis (enter) mean FSS and summarized PDQ were entered as dependent variables and EDSS (0-6.5), disease course (relapsing/progressive forms), time since onset and heat sensitivity age and sex (female/male) were independent variables. In this study, and due to the number of statistical analyses, a *P*-value < 0.010 has been considered a significant value.

### Ethical considerations

The study was guided by common ethical principals in research and according to the Declaration of Helsinki [27]. Approval to use the SwMS register was received from the responsible local administrator. The Regional Ethical Review Board at the Faculty of Health Sciences, Linköping University, Sweden (Dnr M13-07) also approved the study. A completed and returned questionnaire was regarded as granted informed consent.

## Results

### Study group

Two hundred and fifty-six patients, 195 (76%) women and 61 (24%) men, aged 65 years or younger and with an EDSS score between 0 and 6.5, answered the questionnaires. The response rate was 79.3%. Between participants and non-participants there were no statistically significant differences regarding sex (*P *= 0.052), disease course (*P *= 0.664) or EDSS score (*P *= 0.142). The non-participants were about five years younger than the participants, m = 42.7 years, (SD 10.5) (*P *< 0.001) and also had fewer years since onset, Md = 10 (Q_1 _= 5.75; Q_3 _= 15.25) (*P *= 0.014) (Table 1).

**Table 1:** Characteristics of the participants (n = 256)

Age, years, Mean (SD)			47 (SD±11)
*Women*			48.3 (SD±11)
*Men*			44.8 (SD±10.1)
			(*P*=0.022)
Civil status, n (%)			
*Married/cohabitant*			184 (72)
*Living alone*			72 (28)
	
Education, n (%)			
*Lower: Compulsory - Secondary school*			168 (66)
*Higher: After secondary school - University*			88 (34)
	
Occupation, n (%)			
*Work*			106 (42)
*Work and part-time retired or on sick leave*			46 (18)
*No work*			103 (40)
*Missing data (n = 1)*			
Age at onset, year, Md (Q_1 _; Q_3 _)			31 (25; 38)
Years since onset of MS symptoms, Md (Q_1 _; Q_3 _)			13 (8; 22)
	
Course of the disease, n (%)			
*Relapsing - remitting (RR)*			186 (73)
*Progressive forms (primary [PP] and secondary progressive [SP])*			70 (27)
	
Disease severity, n (%)			
*Normal neurological condition, EDSS = *0			26 (10)
*Mild*, 1.0≤EDSS≤ 3.5			157 (61)
*Moderate*, 4.0≤ EDSS≤5.5			30 (12)
*Severe*, 6.0≤ EDSS≤6.5			43 (17)
	
Medication, n (%)			
*No pharmacological treatment*	54 (21)		
*Pharmacological treatment*	202 (79)		
*Immune-modulating treatment*		144 (56)	
*β-interferon*			91 (36)
*glatiramer acetate*			33 (13)
*natalizumab*			20 (8)
*For fatigue*		32 (13)	
*modafinil*			32(13)
*amantadinchlorid*			2(0.7)
*Antidepressants*		46 (18)	
*Analgesics*		61 (24)	
*Spasmolytical treatment*		36 (14)	

Of the participants, the women were somewhat older (*P *= 0.022) than the men, 48.3 (SD 11) and 44.8 (SD 10.1), respectively. At onset the median age was 31 years (Q_1 _= 25; Q_3 _= 38). In 5.5% of the participants, onset occurred before 20 years of age, and the youngest had been 12 years old. The main course of the disease was relapsing-remitting (73%). Ten percent had an EDSS score of 0, but most of the patients (61%) had mild disease severity according to the EDSS. Two hundred and two participants (79%) were being treated pharmacologically. One hundred and forty-four participants (56%) were being treated with immune modulating medication and 32 participants (13%) with medication for fatigue (Table 1).

### Heat sensitivity

One hundred and forty-nine participants (58%) reported heat sensitivity, with no difference between women and men (*P *= 0.102) or in relation to disease course (*P *= 0.226). In relation to EDSS (Table 1), eight of the participants (31%) with normal neurological condition, 98 (62%) with mild disability, 16 (53%) with moderate disability and 27 (63%) with a severe disability reported heat sensitivity. Of those who were sensitive to heat, 70% preferred a room temperature of less than 20 C°, while 73% of those who were not heat sensitive preferred a room temperature of more than 20°C (*P *< 0.001). Eleven of the heat-sensitive participants (n = 149) annotated in the questionnaire that they also were 'cold'.

Of common MS-related symptoms, the heat-sensitive participants reported a significantly higher occurrence of several symptoms than did those who were not heat sensitive, for example fatigue, weakness in legs, concentration difficulties, pain, and urination urgency (Table 2). Weakness in arms and legs as well as balance problems correlated significantly with the EDSS, r = 0.17 (p < 0.010), r = 0.49 (p < 0.010), r = 0.51 (p < 0.010), respectively.

**Table 2:** Significant dichotomized MS symptoms (never to sometimes and usually to always) in relation to reported heat sensitivity (n = 256)

MS-related symptoms*	Appear usually to always n (%)	Heat sensitive %	P-value
Fatigue	148 (58.5)	68	p<0.001
Leg weakness	144 (57)	66	p=0.003
Concentration difficulties	80 (31)	77.5	p<0.001
Loneliness	31 (12)	81	p=0.010
Pain	74 (29)	78	p<0.001
Paraesthesia	100 (40)	69	p=0.004
Urination urgency	51 (20)	76.5	p=0.004

*) Missing values between two and six

In the logistic regression analysis, heat sensitivity significantly explained several MS symptoms, such as fatigue, concentration difficulties and pain. Two of the symptoms, spasms and balance problems, were explained by the EDSS alone (Table 3) while some were explained by more than one variable. Together with heat sensitivity and the EDSS, increasing age also explained the occurrence of weakness in arms (OR = 1.05, 95%CI = 1.02-1.08, *P *= 0.001) and urination frequency (OR = 1.04, 95%CI = 1.01-1.07, *P *= 0.007). Other interesting findings with regard to heat sensitivity, EDSS, and increasing age were weakness in legs (OR = 1.03, 95%CI = 1.0-1.06, *P *= 0.042) and pain (OR = 1.04, 95%CI = 1.0-1.07, *P *= 0.025). Urination urgency was also explained by gender, female (Or = 0.23, 95%CI = 0.07-0.7, *P *= 0.01).

**Table 3:** Logistic regression analyses of dichotomized common MS symptoms (1 = never to sometimes, 2 = usually to always) as dependent variables, and disability (EDSS 0-6.5) and heat sensitivity (1 = yes/0 = no) as independent variables

MS symptoms	EDSS			Heat sensitivity			**R**^**2 **^**Nagelkerke**
		
	OR	95%Cl	*P*-value	OR	95%Cl	*P*-value	
Fatigue	1.15	0.98-1.32	0.086	2.55	1.48-4.25	<0.001	.136
Leg weakness	1.51	1.26-1.81	<0.001	2.21	1.24-3.93	0.007	.274
Spasms	1.79	1.43-2.22	<0.001	1.65	0.77-3.50	0.194	.232
Balance problems	1.62	1.34-1.94	<0.001	1.48	0.83-2.65	0.181	.285
Concentration difficulties	1.08	0.92-1.28	0.354	3.40	1.85-6.25	<0.001	.123
Pain	1.09	0.92-1.29	0.344	3.55	1.87-6.77	<0.001	.136
Paraesthesia	1.20	1.02-1.41	0.026	2.10	1.21-3.64	0.008	.095
Urination urgency	1.27	1.05-1.54	0.016	2.75	1.28-5.90	0.009	.256

The difference in the occurrence of fatigue was confirmed by the FSS. Of those who were heat sensitive, 63% reported severe fatigue, FSS≥5, while among those who were not heat sensitive 45% reported mild or no fatigue, FSS≤4 (*P *< 0.001) (Table 4). One hundred and ten (57%) of the women (n = 192) and 21 (35%) of the men (n = 60) scored an FSS mean value ≥5, classified as fatigued (*P *= 0.010). About a third of the participants who were heat sensitive had concentration difficulties significantly more often (*P *< 0.001). This difference was confirmed by the PDQ, with Md = 29.5 (Q_1 _= 19; Q_3 _= 38) among the heat sensitive participants and Md = 21 (Q_1 _= 12; Q_3 _= 33) in the non-heat-sensitive participants (*P *< 0.001). In the linear regression analyses, heat sensitivity predicted both fatigue and concentration difficulties (Table 5).

**Table 4:** Fatigue assessed in the Fatigue Severity Scale (FSS)* in relation to reported heat sensitivity (n = 252)**

	Non- fatigue (n = 76)	Borderline fatigue (n = 45)	Severe fatigue (n = 131)	Total n	P-value
Heat sensitive, n (%)	30 (39)	25 (55.5)	94 (72)	149	*p*<0.001
Not heat sensitive, n (%)	46 (61)	20 (44.5)	37 (28)	103	

*FSS≤4 = Non-fatigue; 4 > FSS <5 = Borderline fatigue; FSS≥5 = Severe fatigue;

**Missing value, n = 4

**Table 5:** Linear regression analyses of fatigue (FSS) and perceived deficit questionnaire (PDQ) as dependent variables and EDSS (0-6.5) and heat sensitivity (yes/no) as independent variables

	EDSS		Heat sensitivity	
	
	Beta β	*P*-value	Beta β	*P*-value
Fatigue, FSS mean^a)^	0.235	0.001	0.321	<0.001
Concentration difficulties, PDQ sum^b)^	0.041	0.584	0.215	0.001

a) R 0.421; R^2 ^0.177; Adjusted R^2 ^0.161

b) R 0.239; R   0.057; Adjusted R2 0.038

There was no difference between heat-sensitive and non-heat sensitive participants in the use of immune-modulating drugs or treatment for fatigue. Of those being treated with the immune-modulating beta-interferon, fewer reported severe fatigue, FSS≥5.0, compared to those being treated with glatiramer acetate or natalizumab (*P *= 0.021). Of those who were being treated for fatigue (n = 32) a higher proportion reported severe fatigue, FSS≥5.0 (*P *= 0.016), compared to those not being treated for fatigue.

## Discussion

This is an explorative study aimed at investigating the occurrence of heat sensitivity in a group of individuals diagnosed with MS. Further, it aimed to investigate relations between heat sensitivity and common symptoms in MS, disease course and disability.

The participants were recruited from the Swedish MS register in a local county in eastern Sweden. In the chosen EDSS interval, a greater proportion of the patients had an RR course of the disease and were receiving immune-modulating treatment, with regular follow-up visits at the neurological policlinics once a year. Furthermore, some patients with secondary progressive MS and no treatment are seen by general practitioners. Thus, in this study, there is a selection of patients towards an RR course of the disease and who are treated patients. The EDSS scores used were assessed within a year before the data collection and, thus, there might be some random variation in the group. However, if any changes occurred this should be noted in the registry.

Our results indicate that heat sensitivity is highly correlated with several symptoms commonly reported by MS patients. That heat sensitivity is associated with fatigue is well known and has been reported by several authors [4,6-8]. However, our data also reveal that heat sensitivity is a key factor associated with the occurrence of a wide variety of other MS symptoms, for example pain, concentration problems and urination urgency.

### Heat sensitivity intensifies MS symptoms

The most striking result in this study is that heat sensitivity significantly correlated with - and in the logistic regression analyses, appeared as an explaining factor for - the most incapacitating symptoms of MS, viz. fatigue, concentration problems and pain. This result discloses heat sensitivity as a key clinical factor. Our findings are consistent with a Norwegian study by Nortvedt and co-workers [28] stating that bodily pain and low vitality are important problems of MS with a significant impact on quality of life. Low vitality [28] can be interpreted as strongly associated to the symptom of fatigue.

We found that many other MS symptoms are also correlated with heat sensitivity. Interestingly, similar observations were actually reported in the early works of Uthoff, although subsequent citations narrowed the interpretation of Uthoff's phenomenon to blurring of the vision [6]. In the present study, however, no relationship between the symptom of blurred vision and heat sensitivity was confirmed.

What might be the cause of the observed co-occurrence of heat sensitivity and pain, fatigue and cognitive problems? Can this indicate something about the pathogenic mechanism? Based on studies of post-stroke patients, Craig [29] suggests that central pain might be caused by a thermoregulatory dysfunction. A lesion in the thalamus releases or disinhibits the feeling of burning pain by removing the normal inhibition of pain by cold. The role of the thalamus is as ascending relay of spinothalamic activity to the cortex [29]. This is similar to Österberg's [30] proposal that central pain in MS seems to be generated through lesions affecting the spinothalamo-cortical pathways [30]. A third of the MS patients with central pain had visible lesions in the thalamus [31]. Thus, one can speculate that the thalamus might be involved.

It is also interesting to think about the effects of decreased sweating in terms of electrolytic imbalance and secondary neuronal effects. Recent results by Saari and co-workers [32] disclose such an impairment to thermoregulatory sweating in MS. One idea that may explain thermoregulatory dysfunction is that lesions can affect important cerebral areas such as the hypothalamus. The study by Saari [32] also demonstrated a correlation between increasing sweating impairment and increasing disability (EDSS), which is congruent with our results showing a correlation between heat sensitivity and increasing EDSS.

Fatigue in MS has been eagerly studied by many researchers during the past decade. Fatigue and heat sensitivity are related in many aspects, and a subsequent question is whether they have common pathogenic features. Recently, Marino [6] stated that MS fatigue is likely to be a central rather than a peripheral phenomenon. Heat sensitivity in MS, however, is not understood as clearly.

### The mechanism behind heat sensitivity

The mechanism of heat sensitivity in MS is reviewed in a recent article by Marino [6]. The heat reaction blocks the action potential of the demyelinated neuron (frequency-dependent conduction block, - FDCB) [3]. The demyelinization results in a slower nerve conduction velocity. A conduction block can occur because the damaged axons transmit only single or low frequency impulses, instead of high frequency impulse trains like in healthy nerve tissue [33]. Marino [6] states that this observation is very important, especially when an increase in temperature blocks nerve impulses in demyelinated fibres [34]. Interestingly, very small increases in temperature can also block action potentials [6]. The heat sensitivity in MS is described by Baker as secondary to both environmental heat and environmental humidity, as well as to exercise [35]. Both passive and active body temperature increases give heat reactions. Clinical reports from individual patients in our MS clinic (i.e. outside this study) reveal that their experience of temperature aberrations can vary greatly, indicating that the mechanisms may be multiple.

### The subjective phenomenon of heat sensitivity

Many patients with MS are aware of their heat sensitivity, and have experienced increased clinical symptoms when becoming warm from fever, hot environmental temperature or physical exercise. In this study, patients who did or did not report heat sensitivity were dichotomized and analysed according to this categorization. It is worth noting that some patients reported both heat sensitivity and a subjective feeling of "cold". This suggests that temperature aberrations in MS can be complex and individual. In our clinical practice we also encounter MS patients with complex subjective temperature aberrations, e.g. patients who deny heat sensitivity when asked, yet prefer to sleep in cold bedrooms because it alleviates their MS symptoms. Such patients may not yet be aware of a mild heat sensitivity. This factor also suggests that milder forms of heat sensitivity may be underreported in a study like ours. We have also met one patient who likes to be in the sun all day without any side effects, but after jogging has to take repeated showers to prevent incapacitating fatigue. Finally, it is well known that patients with severe MS can sometimes develop hypothermia, often without any major subjective symptoms. We suggest that qualitative studies should be initiated to describe patients' experiences of subjective phenomena like heat sensitivity, and feeling cold as well as combinations of the two. One cannot exclude that central and peripheral mechanisms interact in the "temperature syndrome" of MS, which includes more features than classical heat sensitivity.

### Fatigue as a side effect of immune modulating drugs

It has recently been suggested that beta-interferon can increase fatigue through influenza-like side effects including hyperthermia, but that glatiramer-acetate, on the other hand, is a neutral agent in this respect, and that natalizumab at least in some cases decreases fatigue. Some scientific reports support these observations [16-18]. In our study, however, none of these relationships could be demonstrated. However, as this study has a descriptive design, no conclusion regarding what impact immune modulating treatment has on fatigue could be drawn.

## Conclusions

In conclusion, although heat sensitivity in MS was described as early as the late 19^th ^century and is a well-known phenomenon today, it has to date been disregarded in studies of fatigue in MS [6]. The findings in this study underline the importance of heat sensitivity in MS patients as a key symptom that is highly correlated with disabling symptoms such as fatigue, concentration difficulty and urination urgency. A majority of the participants rated the symptom of fatigue as their most impairing symptom. Furthermore, a significantly higher proportion among the heat-sensitive participants rated higher levels of fatigue compared to the participants who were not heat sensitive.

The results of our study put heat sensitivity in the position of a key clinical symptom. Our findings emphasize the need to further investigate the mechanism of heat sensitivity: What is the role of the sweating impairment? Is there thalamic involvement? What is the role of the immune system? One should also analyse what it means for patients and in the care of MS patients. Finally, a challenging topic to investigate is how heat sensitivity can be treated clinically, for example using thermotherapy.

## Competing interests

The authors declare that they have no competing interests.

## Authors' contributions

All authors contributed to the planning of the study. GF collected all data and, together with A-CE, conducted the statistical analyses. In collaboration, GF, A-CE and A-ML wrote the drafts, which were repeatedly read, discussed and revised by all the authors. Finally, all authors read and approved the final manuscript before its submission.

## Pre-publication history

The pre-publication history for this paper can be accessed here:

http://www.biomedcentral.com/1471-2377/11/27/prepub
